# Cost-effectiveness analysis of durvalumab combined with tremelimumab versus sorafenib as first-line treatment for unresectable Hepatocellular carcinoma in China: a dual-model approach utilizing partitioned survival and markov models

**DOI:** 10.1186/s12876-026-05305-3

**Published:** 2026-09-07

**Authors:** Ruixuan Liu, Yong Xiao, Zhen Wang, Qing Yang

**Affiliations:** 1 School of Nursing, Chengdu University of Traditional Chinese Medicine, No. 1166, Liutai Avenue, Wenjiang District, Chengdu, 611137 Sichuan China; 2 Department of Nursing, Sichuan Clinical Research Center for Cancer, Sichuan Cancer Hospital & Institute, Sichuan Cancer Center, University of Electronic Science and Technology of China, Chengdu, China

**Keywords:** Cost-effectiveness analysis, First-line systemic therapy, Unresectable hepatocellular carcinoma, Markov model, Partitioned survival model

## Abstract

**Background:**

Hepatocellular carcinoma (HCC) is a major contributor to global cancer-related mortality, with the majority of patients diagnosed at unresectable stages. The STRIDE regimen (tremelimumab plus durvalumab) demonstrated superior survival benefits over sorafenib in the HIMALAYA trial. However, its cost-effectiveness within the Chinese healthcare system remains unverified.

**Objective:**

To evaluate the cost-effectiveness of first-line STRIDE versus sorafenib for unresectable HCC from the perspective of the Chinese healthcare payer.

**Methods:**

A comprehensive cost-effectiveness analysis was conducted using both partitioned survival (PartSA) and Markov state-transition models over a 10-year time horizon. Clinical efficacy parameters were derived from reconstructed HIMALAYA trial survival data. Direct medical costs were estimated based on post-negotiation drug pricing in China and published sources. Health utility weights were sourced from established literature. Scenario analyses (50% price reduction, 0% discount rate) and comprehensive deterministic and probabilistic sensitivity analyses (PSA) were performed to evaluate model uncertainty.

**Results:**

In the base-case analysis, the STRIDE regimen yielded incremental cost-effectiveness ratios (ICERs) of $204,713.55/QALY in the PartSA model and $208,385.86/QALY in the Markov model, both substantially exceeding China’s willingness-to-pay threshold (3× GDP per capita, $36,975/QALY). Under a 50% price reduction scenario, the ICER dropped to $87,351.95/QALY but remained non-cost-effective. Probabilistic sensitivity analysis demonstrated a < 20% likelihood of STRIDE being cost-effective across tested scenarios. Key cost drivers included drug acquisition costs and progression-free utility values.

**Conclusion:**

Under current pricing, the STRIDE regimen is not cost-effective as a first-line treatment for unresectable HCC in China. While price concessions improve affordability, exploration of value-based pricing and future biomarker-guided patient stratification could be evaluated to optimize economic viability. The dual-model concordance reinforces the robustness of these findings.

**Supplementary Information:**

The online version contains supplementary material available at https://doi.org/10.1186/s12876-026-05305-3.

## Article Key points

- Under current pricing in China, first-line STRIDE for unresectable HCC is not cost-effective, with base-case ICERs ($204,714/QALY) far exceeding the willingness-to-pay threshold ($36,975/QALY).

- A 50% price reduction substantially lowers the ICER to $87,352/QALY, yet additional value-based price negotiations and real-world stratified evaluations are necessary.

- Dual-model validation (PartSA and Markov) yields highly concordant results, ensuring structural robustness against modeling biases.

**Supplementary Information:**

The online version contains supplementary material available at https://doi.org/10.1186/s12876-026-05305-3.

## Introduction

Hepatocellular carcinoma (HCC) constitutes the predominant histological subtype of liver cancer, accounting for > 80% of primary cases globally [1]. As the third leading cause of cancer-related mortality worldwide, it poses a substantial public health challenge. While curative interventions (e.g., resection, ablation, or transplantation) are feasible for early-stage HCC, > 70% of patients present with unresectable disease at diagnosis, resulting in poor prognoses and limited therapeutic options.

The HIMALAYA phase III trial (NCT03298451) [2] (First Posted on 02 October 2017) at ClinicalTrials.gov. marked a pivotal advancement in first-line systemic therapy for unresectable HCC. This landmark study demonstrated that the STRIDE regimen (Single Tremelimumab Regular Interval Durvalumab), a novel combination comprising the anti-CTLA-4 antibody tremelimumab and the anti-PD-L1 antibody durvalumab, significantly improved overall survival (OS) and progression-free survival (PFS) compared to sorafenib, the long-standing standard-of-care targeted therapy (multikinase inhibitor) [3]. Notably, both regimens exhibited comparable safety profiles, with similar incidences of any-grade adverse events (97.4% for STRIDE vs. 95.5% for sorafenib). These results establish STRIDE as a clinically compelling first-line alternative for HCC.

China bears a disproportionate burden of HCC, with 367,700 new cases and 316,500 deaths reported in 2022, representing the fourth-highest incidence and second-highest mortality globally [4–6]. This epidemic imposes immense pressure on China’s healthcare resources and socioeconomic development. In response, the 2017 inclusion of sorafenib in China’s National Reimbursement Drug List (NRDL) achieved substantial cost reduction: out-of-pocket expenses decreased from tens of thousands to several thousand RMB per package, significantly enhancing patient affordability [7]. Despite this policy advancement, persistent financial toxicity in cancer care remains a critical barrier, especially for high-cost innovative biologic agents [8].

Against this backdrop, the cost-effectiveness of emerging therapies must be rigorously evaluated to inform sustainable resource allocation [9]. Leveraging contemporary clinical trial evidence, updated drug pricing, and current clinical guidelines, we developed and calibrated both a partitioned survival model and a Markov model to evaluate the cost-effectiveness of tremelimumab plus durvalumab from a Chinese healthcare payer perspective [10]. This analysis provides evidence-based insights to inform future healthcare policy reform.

## Materials and methods

### Population and intervention

A total of 393 patients were assigned to receive the STRIDE regimen based on the HIMALAYA study, and 389 patients received sorafenib. The STRIDE regimen consisted of a single dose of 300 mg of tremelimumab plus 1,500 mg of durvalumab every four weeks. Sorafenib was given at a dose of 400 mg twice daily. Tumours were assessed using RECIST v1.1 at baseline and every eight weeks for the first 48 weeks after randomisation, and then every 12 weeks until confirmed progression, via computed tomography or magnetic resonance imaging. Patient-reported outcomes were evaluated using the European Organisation for Research and Treatment of Cancer 30-Item Quality of Life Questionnaire [11]. Treatment continued until progression, unacceptable toxicity, withdrawal of consent, or other discontinuation criteria were met. Following discontinuation of treatment, 40.7% and 45.0% of patients in the STRIDE and sorafenib groups, respectively, received subsequent second-line anticancer therapy.

### Model structure

**Fig. 1:**
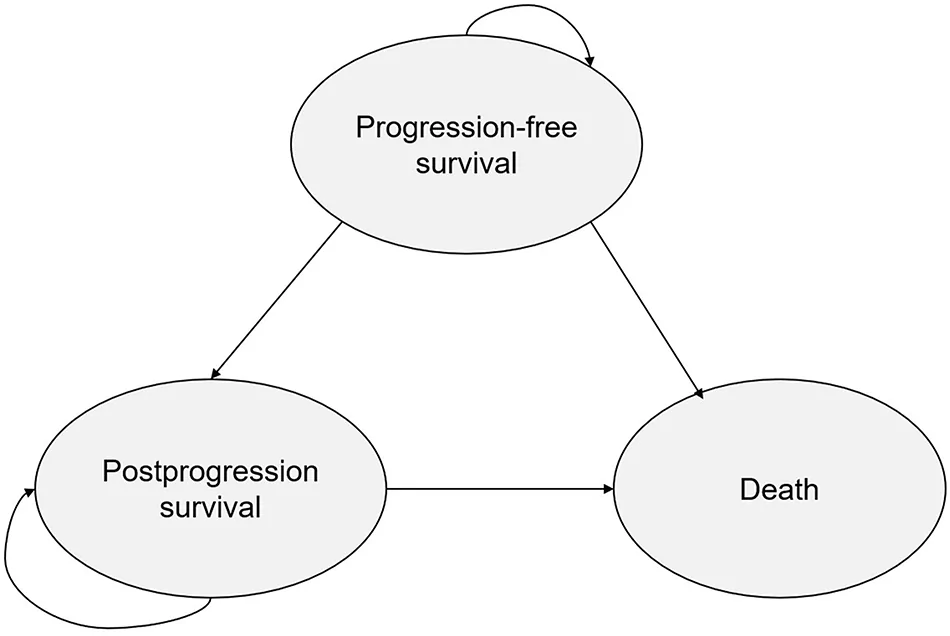
Cohort-based model with 3 states

In this study, to address potential model uncertainty, we used two models to derive our results. PartSA and Markov models were constructed using Treeage Pro Healthcare 2022 software. The simulated cohort followed the natural history of advanced HCC through three mutually exclusive health states: progression-free survival (PFS), disease progression (PD), and death (Death) [12]. All simulated patients started in the progression-free survival state, transitioned through the healthy state, and ended in the death state (Fig. 1). In the Markov model, the time-dependent transition probabilities between states were calculated using survival curve parameters. The natural death probability was assumed to be the probability of transitioning from PFS to death, with a half-cycle correction applied; In the ParSA model, the proportion of patients surviving for t cycles is estimated using the area under the OS curve, the proportion of patients without disease progression is estimated using the area under the PFS curve, and the proportion of patients with disease progression is estimated based on the difference between the OS and PFS curves. In the study, the probability of death without disease progression in the model is assumed to be the natural mortality rate [13]. According to the treatment plan, the treatment cycle is assumed to be one month. The discount rate for all costs and quality-adjusted life years (QALYs) was 5% per year, and the simulation study spanned 10 years (120 cycles, during which most patients died). The willingness-to-pay (WTP) threshold was set at three times China’s 2023 per capita gross domestic product (GDP), which is $36,975 [14]. The primary outcomes of our study include total costs, incremental cost-effectiveness ratio (ICER), quality-adjusted life years (QALYs), life years (LYs), incremental net health benefit (INHB), and incremental net monetary benefit (INMB) [15]. This cost-effectiveness analysis was performed according to the Consolidated Health Economic Evaluation Reporting Standards (CHEERS) guidelines [16].

### Clinical data

Use the GetData Graph Digitizer software to extract numerical points from the OS and PFS curves reported in HIMALAYA, and use the SurvHE package in R 4.4.3 software to reconstruct individual data [17, 18]. HIMALAYA is a randomized, open-label, multicenter Phase 3 trial designed to evaluate the efficacy of the STRIDE regimen versus sorafenib treatment in patients with unresectable hepatocellular carcinoma who have not previously received systemic therapy.

**Fig. 2:**
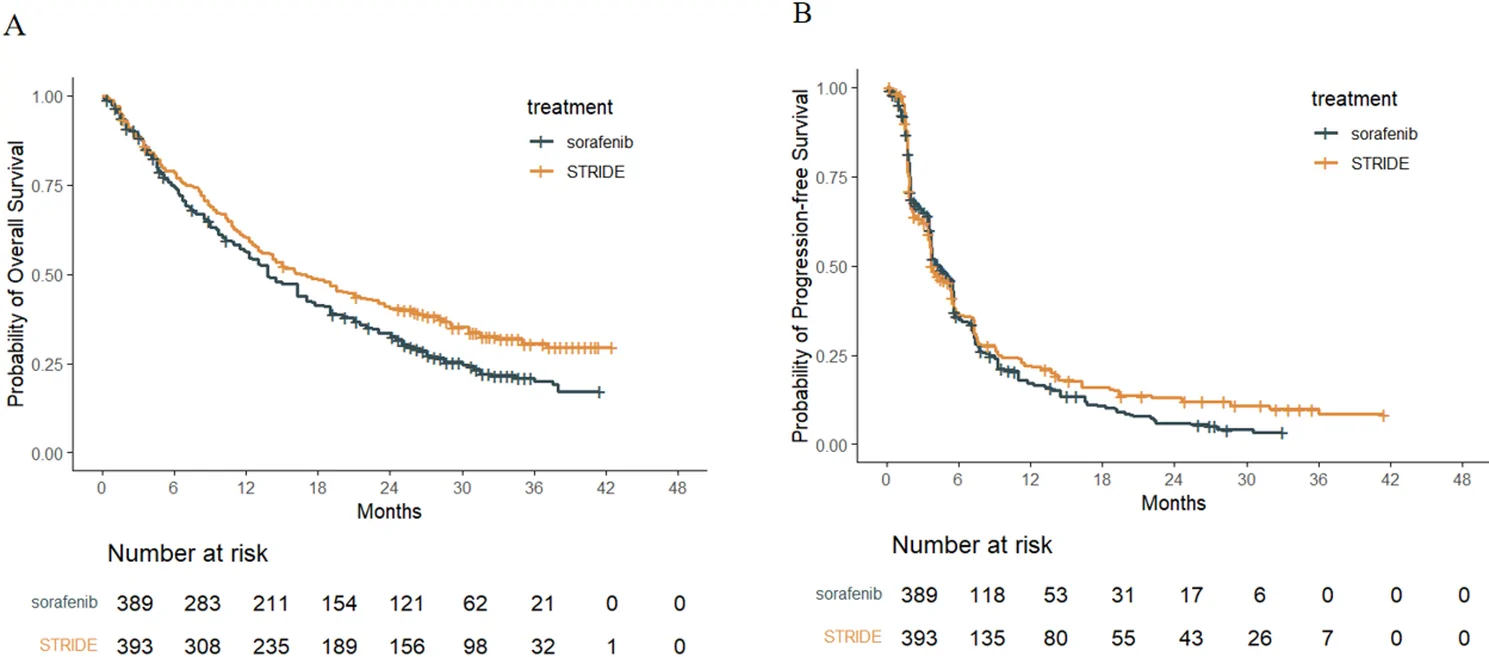
Reconstruction of Kaplan-Meier survival curves. (**A**) OS curve (**B**) PFS curve

Because immunotherapy may generate prolonged survival tails, several parametric survival functions were explored to extrapolate long-term outcomes beyond the trial follow-up period. We parameterized the reconstructed data using five survival distributions (exponential distribution, Weibull distribution, Compertz distribution, log-logistic distribution, and log-normal distribution) and ultimately assessed the median survival time of the Kaplan-Meier curves [19, 20]. Based on goodness-of-fit tests, namely the Akaike information criterion (AIC) and Bayesian information criterion (BIC), combined with visual inspection, the function corresponding to the best-fitting distribution was determined [21]. The survival model results for the STRIDE group and sorafenib group are shown in Table 1, and the good visual fit of the data is shown in Fig. 2. The survival events and survival times of the virtual individual patients were similar to those of the actual risk population, and this was closely reproduced in the survival curves. The optimal fitting distribution for the OS and PFS curves in both the STRIDE group and sorafenib group was the log-normal distribution.

**Table 1:** Results of fitting to the observed data

		Exponential	Weibull	Gompertz	Log-normal	Log-logistic
STRIDE OS	AIC	2261.33	2262	2254.01	2241.84	2247.86
	BIC	2265.30	2269.95	2261.96	2249.79	2255.81
STRIDE PFS	AIC	2175.354	2162.875	2096.008	2051.776	2054.008
	BIC	2179.328	2170.823	2103.955	2059.723	2061.956
Sorafenib OS	AIC	2369.29	2371.2	2370.34	2366.58	2374.12
	BIC	2373.25	2379.12	2378.27	2374.51	2382.05
Sorafenib PFS	AIC	2015.429	2016.619	2009.07	1958.959	1959.601
	BIC	2019.393	2024.546	2016.997	1966.886	1967.528

### Markov transition probabilities

Time-dependent transition probabilities in this three-state Markov model were calculated based on KM survival curves for PFS and OS for each treatment. Take the STRIDE group as an example, we got the value of µ and σ from the log-normal distribution for the PFS curve and the OS curve. We defined them µPFS, σPFS, µOS, and σOS. The probabilities for patients in the PFS state to keep in the same state (pFTF) were calculated by using µPFS and σPFS. Due to the absence of granular individual patient data regarding cause-specific pre-progression mortality in published trial reports, the transition probability from the progression-free state directly to death (pFTD) was assumed to reflect age-specific background natural mortality. Consequently, the transition probability from the progression-free state to the post-progression state (pFTP) was calculated as 1-pFTF-pFTD. Although this background mortality assumption is conventional in oncology decision-analytic modeling, we recognize that it may underestimate early disease-related liver failure before radiographic progression; this structural limitation was cross-validated using the parallel PartSA framework. Similarly, according to the µOS and σOS, we got the probabilities from the survival states (including the PF state and PP state) to the survival states (pSTS) and the probabilities from the survival states to death (pSTD). Then we could strip the probabilities from the post-progression state to the post-progression state (pPTP) from the pSTS. The formula was following [22]:

1 $$\begin{array}{lll}\mathrm{pPTP}_{\mathrm{n}} &=& [\mathrm{(PFS}_{\mathrm{n-1}}+\mathrm{PD}_{\mathrm{n-1}})\\&&*\mathrm{pSTSn-PFS}_{\mathrm{n-1}}*\mathrm{pFTF}_{\mathrm{n}}\\&&-\,\mathrm{PFS}_{\mathrm{n-1}}*\mathrm{PFTP}_{\mathrm{n}}]/ \mathrm{PD}_{\mathrm{n-1}}\end{array}$$

In this formula, the PFS_*n*−1_ was defined as the patients in the PF state in n-1 cycle, and PD_*n*−1_ was defined as the patients in the post-progression state in n-1 cycle. The probabilities from the post-progression state to the death state was1-pPTP_n_.

### Cost and utility estimate

This study only includes direct medical costs within the healthcare system, including the cost of anticancer drugs, the cost of efficacy and toxicity testing required during treatment, drug management, follow-up treatment after disease progression, laboratory tests, and the cost of adverse event (AE) management. We considered the costs of the following adverse reactions: diarrhea, palmar-plantar erythrodysesthesia syndrome, and hypertension, as these side effects were the most common and clinically relevant Grade 3 or 4 events in the trial. The treatment cost for each cycle is calculated based on the dosage schedule and the unit price of the medication (unit price × dosage for one cycle). We utilized data from the Chinese Drug Information Website (www.yaozh.com), with relevant costs derived from published literature [21]. All costs were converted to USD at an exchange rate of 1 USD = 7.28 RMB (as of March 1, 2025), with each item’s cost collected as of the relatively stable date of March 1, 2025. Health state utility scores were derived from previously published literature: 0.76 for the progression-free state and 0.68 for the progressed disease [15, 23, 24] (Table 2).

**Table 2:** Model parameter baseline values, and distributions for sensitivity

Variables	Baseline Value (±20%)	Distribution	Reference
Costs input ($)		Gamma	
Sorafenib per cycle	1379.84 (1103.87-1655.8)	Gamma	yaozh.com
tremelimumab per cycle	12380.9524 (9904.76-14857.14)	Gamma	[25]
Durvalumab per cycle	7488 (5990.4-8985.6)	Gamma	yaozh.com
Test	175.4512 (140.36-210.54)	Gamma	[25]
AE	12.4781 (9.982–14.973)	Gamma	[25]
second-line therapy	937.3418 (749.87-1124.81)	Gamma	[25]
Parameter			
Lognormal OS survival model of STRIDE	µ = 2.8613 σ = 1.4121	Lognormal	Model fitting
Lognormal PFS survival model of STRIDE	µ = 1.6168 σ = 1.143	Lognormal	Model fitting
Lognormal OS survival model of Sorafenib	µ = 2.5905 σ = 1.3126	Lognormal	Model fitting
Lognormal PFS survival model of Sorafenib	µ = 1.5149 σ = 1.0246	Lognormal	Model fitting
Grade 3–4 AE probabilities in tremelimumab plus durvalumab arm, %			
Diarrhea	4.4(3.08–5.72)	Beta	[2]
Palmar–plantar erythrodysesthesia syndrome per event	0	Beta	[2]
Hypertension	1.8(1.26–2.34)	Beta	[2]
Grade 3–4 AE probabilities in sorafenib arm, %			
Diarrhea	4.3(3.01–5.59)	Beta	[2]
Palmar–plantar erythrodysesthesia syndrome per event	9.1(6.37–11.83 )	Beta	[2]
Hypertension	6.1(4.27–7.93)	Beta	[2]
Any subsequent second-line therapy, %			
Durvalumab plus tremelimumab	40.7	Beta	[2]
Sorafenib	45	Beta	[2]
Utility estimate			
utility in PFS	0.76	Beta	[15, 23, 24]
utility in PD	0.68	Beta	[15, 23, 24]
utility in Death	0	Beta	

### Sensitivity analyses

In this study, we conducted one-way and probabilistic sensitivity analyses to estimate the robustness of the model results. One-way sensitivity analyses were performed for different variables (including costs and utilities), with each variable’s uncertainty assumption varying by 20% from the baseline value [26]. Probability sensitivity analysis was performed using Monte Carlo simulation with 10,000 iterations to test the uncertainty of the model with pre-specified probability distributions [27]. The Gamma distribution was used for cost parameters, and the Beta distribution was selected for probability, proportion, and preference value parameters [28]. Based on data from 1,000 iterations, scatter plots and cost-effectiveness acceptability curves were constructed to assess the likelihood of cost-effectiveness advantages across groups at different threshold levels.

### Scenario analysis

This study designed two key scenario analyses to systematically evaluate the cost-effectiveness of the STRIDE regimen. First, based on the observation that the ICER in the current baseline analysis exceeded China’s conventional willingness-to-pay threshold, we designed a scenario simulation with a 50% price reduction to examine the potential cost-optimization effects of national medical insurance negotiations or centralized bulk procurement. Second, to assess the impact of time preference factors on economic evaluation, we specifically designed an analysis scenario with a discount rate of 0% to comprehensively examine the economic value of the long-term survival benefits of this immunotherapy regimen. These two scenario analyses provide a multi-dimensional research framework for the economic evaluation of the STRIDE regimen from different dimensions (price policy factors and time preference factors).

## Results

### Base-case analysis

In the PartSA model, the total cost for the STRIDE group was $80,201.03, with a benefit of 1.74 QALYs, while the total cost for sorafenib was $10,750.41, with a benefit of 1.40 QALYs. The ICER for STRIDE compared to sorafenib is $204,713.55/ QALY; in the Markov model, the ICER is $208,385.86 /QALY. At a WTP threshold of three times the per capita GDP, STRIDE is not cost-effective. (Table 3)

**Table 3:** Base-case results

Strategies	PartSA Model				Markov Model			
	Total cost	LYs	QALYs	ICER($/QALY)	Total cost	LYs	QALYs	ICER($/QALY)
STRIDE	80201.03	2.47	1.74	204713.55	78070.40	2.31	1.76	208385.86
sorafenib	10750.42	1.98	1.40		10642.61	1.89	1.43	

### Deterministic sensitivity analyses

**Fig. 3:**
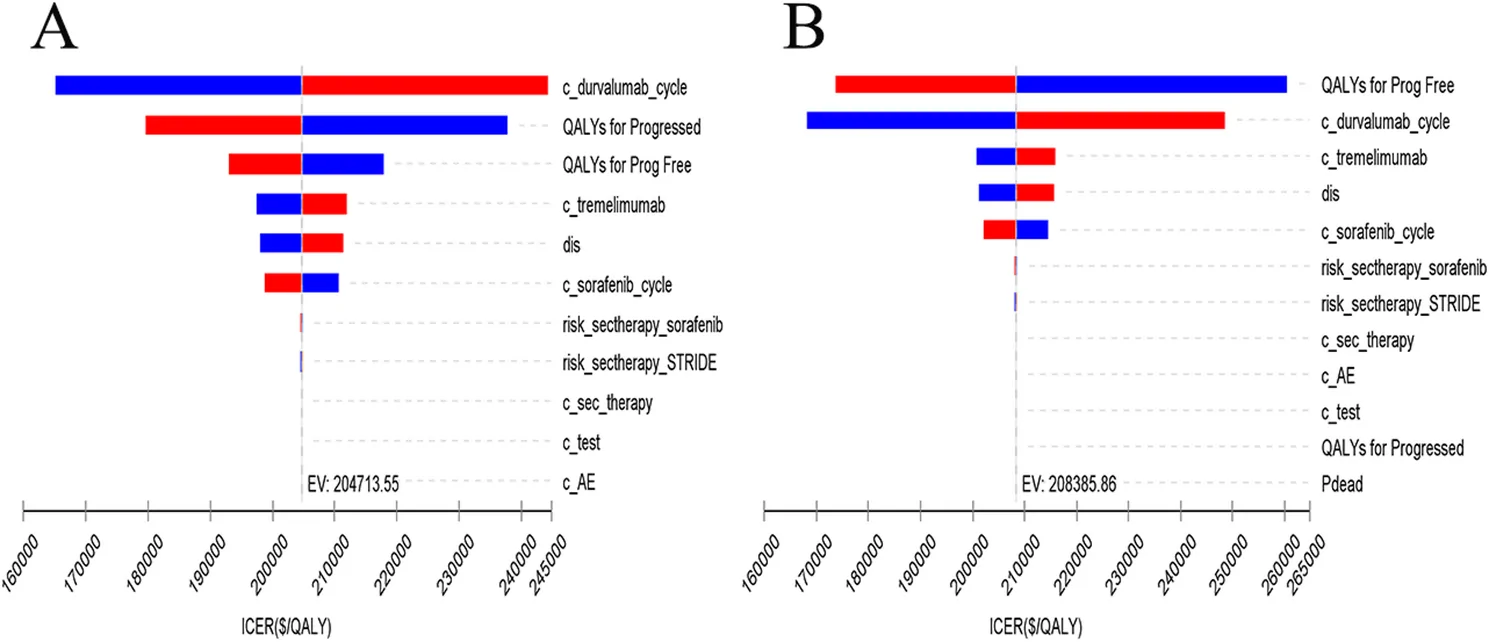
Toronto diagram of one-way sensitivity analysis. (**A**) PartSA model. (**B**) Markov mode

The tornado plot indicates that in the PartSA model, the cost of Durvalumab, the utility value of PFS, and the utility value of PD have a significant impact on the ICER; in the Markov model, the utility value of PFS, the cost of Durvalumab and tremelimumab, and the discount rate have a significant impact on the ICER. Across a range of parameters, the results remain robust regardless of parameter changes, with the ICER consistently exceeding the willingness-to-pay (WTP) threshold. Therefore, STRIDE is not cost-effective compared to sorafenib targeted therapy (Fig. 3).

### Probabilistic sensitivity analysis

**Fig. 4:**
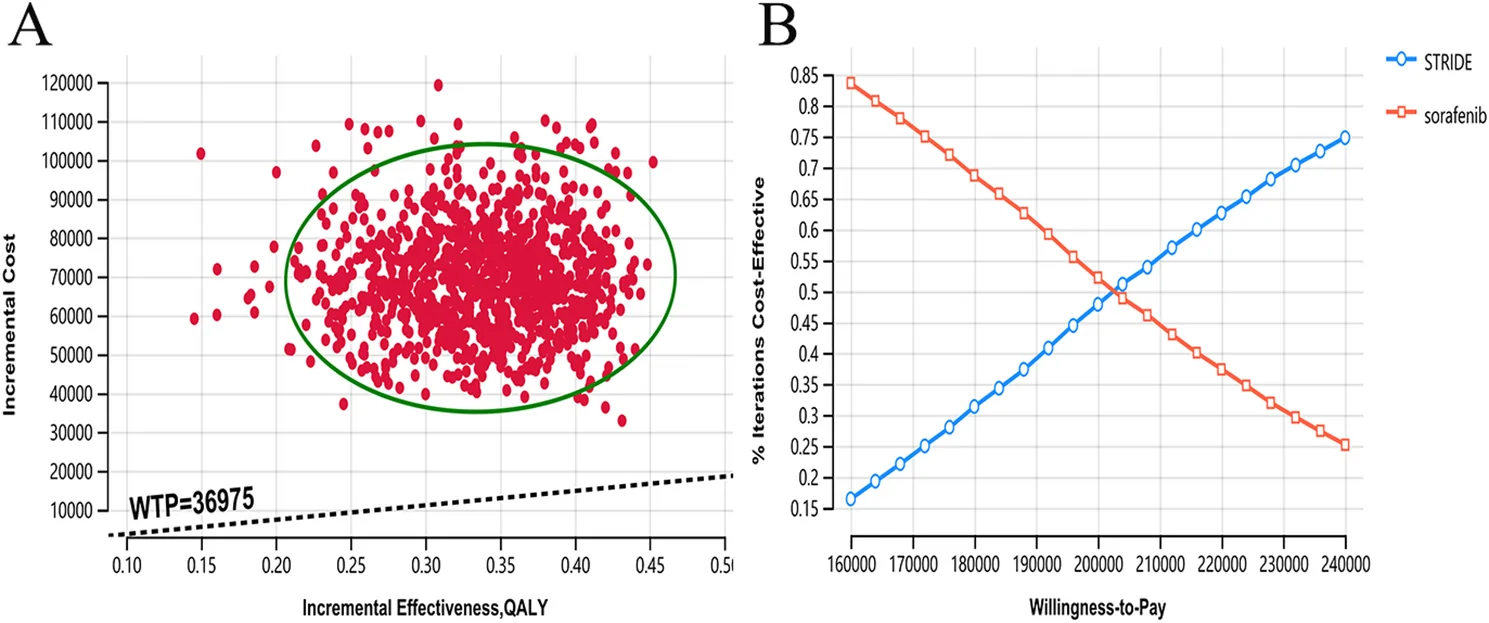
(**A**) The scatterplot. (**B**) Cost-effectiveness Acceptability Curves for STRIDE vs Sorafenib.STRIDE, tremelimumab and durvalumab combination; WTP, willingness-to-pay

The cost-effectiveness scatter plot and cost-effectiveness acceptability curve for probability sensitivity analysis are shown in Fig. 4A and B, respectively. Figure 4A shows that all data points are located in the first quadrant, indicating that the STRIDE regimen is more effective than the sorafenib regimen in treating unresectable hepatocellular carcinoma, but at a higher cost; more than half of the scatter points are distributed above the WTP threshold curve at three times China’s per capita GDP, indicating that the STRIDE regimen is not cost-effective. Figure 4B shows that as WTP increases, the acceptable proportion for sorafenib remains at 100%. When WTP exceeds $200,000 /QALY, the acceptable proportion decreases. However, when the WTP threshold exceeds $210,000 /QALY, the STRIDE strategy achieves the highest cost-effectiveness.

### Scenario analysis

The study results indicate that the STRIDE program failed to meet China’s cost-effectiveness threshold standard under both scenario analyses. In the scenario where prices decreased by 50%, the total treatment cost of the STRIDE program decreased from the baseline of $80,201.03 to $40,385.23, with the corresponding ICER value decreasing from $204,713.55 per QALY to $87,351.95 per QALY (a 42% reduction), but it remains significantly higher than China’s payment threshold of three times the per capita GDP ($36,975). Probability sensitivity analysis indicates that the probability of the STRIDE regimen being cost-effective in this scenario is less than 20%. In the scenario with a discount rate of 0%, although the health outcomes of the STRIDE regimen improved to 2.00 QALYs (compared to 1.58 QALYs for sorafenib), the ICER value remained at a high level of $172,852.91 per QALY due to the high treatment cost of $84,190.44 (an increase of $73,014.25 compared to sorafenib). Both analyses consistently indicate that, under the current pricing system, the economic viability of the STRIDE regimen in China’s healthcare system remains significantly challenged, even when considering price regulation or long-term survival benefits.(Table 4).

**Table 4:** Scenario analyses results

Strategies	Scenario 1			Scenario 2			
	Total cost	QALYs	ICER($/QALY)	Total cost		QALYs	ICER($/QALY)
STRIDE	40385.23	1.74	87351.95	84190.44	2.00		172852.91
sorafenib	10750.41	1.40		11176.19	1.58		

## Discussion

This study represents, to our knowledge, the first cost-effectiveness analysis comparing the tremelimumab plus durvalumab (STRIDE) regimen versus sorafenib as first-line therapy for unresectable hepatocellular carcinoma (uHCC) within the Chinese healthcare context. Adopting a Chinese healthcare system perspective, our analysis incorporated post-negotiation national drug pricing for all agents and adjusted other medical costs using locally sourced data. This approach enhances the relevance of our findings to reimbursement decision-making in China’s evolving healthcare landscape [26].

To ensure methodological robustness, we employed dual modeling frameworks: a Markov cohort model and a partitioned survival model [29]. Notably, both models yielded closely aligned incremental cost-effectiveness ratios (ICERs), reinforcing the consistency and reliability of our conclusions. This concordance across structurally distinct models mitigates concerns regarding model-specific biases. Our comprehensive sensitivity analyses identified several key drivers of cost-effectiveness: uility weights for progression-free health states, acquisition costs of durvalumab and tremelimumab and discount rate. It is noteworthy that participants taking STRIDE had a lower relative risk of decline in quality of life compared to participants taking sorafenib [30].The substantial acquisition cost of tremelimumab emerged as a particularly critical barrier. As a novel biologic agent, its current pricing structure poses a significant challenge to cost-effectiveness attainment. This economic burden may substantially compromise patient accessibility and affordability, potentially limiting real-world adoption despite demonstrated clinical efficacy [31]. When evaluated against conventional willingness-to-pay (WTP) thresholds in China (typically 1–3 times GDP per capita), the STRIDE regimen consistently failed to demonstrate cost-effectiveness. The primary economic disadvantage stems from its premium pricing relative to sorafenib targeted monotherapy, which is not sufficiently offset by incremental health gains within our model projections.

The results of our scenario analysis demonstrate that the STRIDE regimen fails to meet China’s conventional willingness-to-pay threshold even under a substantial 50% price reduction. This finding naturally prompts consideration of whether precision medicine strategies, such as biomarker-guided patient stratification, could enhance the economic viability of high-cost cancer immunotherapies. From a health policy perspective, implementing tiered reimbursement targeted at high-value patient subgroups represents an appealing conceptual route to maximize value-based resource allocation while containing overall healthcare expenditures [32, 33]. However, it must be emphasized that in the absence of biomarker-stratified clinical efficacy data (such as PD-L1 expression subgroups) from the pivotal HIMALAYA trial and corresponding quantified subgroup ICER models, such reimbursement proposals currently remain an exploratory hypothesis rather than an actionable conclusion. Unlike non-small cell lung cancer, where biomarker-stratified reimbursement is well established, the predictive utility of PD-L1 expression in advanced hepatocellular carcinoma remains biologically heterogeneous and methodologically unstandardized. Therefore, dedicated clinical trials and stratified pharmacoeconomic modeling are essential prerequisites to quantitatively substantiate both clinical efficacy and economic sustainability in biomarker-selected HCC populations.

Crucially, translating such a biomarker-guided reimbursement framework into routine clinical practice within China’s healthcare ecosystem presents several pragmatic hurdles. First, standardized PD-L1 immunohistochemistry (IHC) assays exhibit substantial variability across antibody platforms and scoring algorithms (TPS vs. CPS), with advanced diagnostic capabilities largely restricted to tertiary oncology centers rather than regional or primary hospitals. Second, companion diagnostic testing fees are not yet universally covered by the basic medical insurance catalog across all provinces, creating out-of-pocket financial barriers to initial screening. Third, the real-world PD-L1 positivity rate among Chinese HCC patients is relatively modest and variable. To bridge this translational gap, healthcare authorities and policymakers should consider: (1) establishing standardized regional pathology quality control networks led by National Cancer Center-affiliated hubs; (2) incorporating essential companion diagnostics into national medical insurance reimbursement schedules alongside drug pricing negotiations; and (3) advancing multi-omics biomarker panels to enhance predictive accuracy and clinical utility in advanced HCC.

Our 0% discount rate analysis provides unique insights into the time-dependent benefit characteristics of STRIDE, showing that when considering its full long-term survival benefits, quality-adjusted life years (QALYs) can increase by 14.9%. Although this method challenges traditional health technology assessment approaches, it appropriately captures the sustained efficacy characteristic of immunotherapy [34]. Second, despite the increase in health outcomes, the ICER remains at a high level $172,852.91/QALY), significantly exceeding China’s payment threshold of three times per capita GDP. This phenomenon indicates that, even without considering time preference factors, the economic challenges of the STRIDE regimen under the current pricing system persist. From a clinical value perspective, the analysis results at a discount rate of 0% reinforce the STRIDE regimen’s advantage in extending patient survival time, consistent with the long-term survival curve characteristics observed in the HIMALAYA clinical trial. However, from a health economics perspective, the persistently high ICER indicates that the health benefits derived solely from extended survival are insufficient to offset the high treatment costs of the scheme. This finding suggests that when evaluating the value of immunotherapy combination regimens, a more comprehensive evaluation framework is needed, one that considers not only extended survival time but also improvements in quality of life, as well as the indirect impacts of treatment on patients’ families and the socioeconomic system. It is worth noting that while the assumption of a discount rate of 0% does not align with conventional economic principles, it holds particular significance for assessing the long-term value of innovative therapies. This analytical approach highlights the true value of treatment regimens that may yield “long-tail effects,” particularly for immunotherapy, which may provide long-term survival benefits [35, 36].

This cost-effectiveness analysis has several methodological limitations that warrant consideration. First, the clinical efficacy inputs were derived exclusively from the single Phase III HIMALAYA trial rather than real-world datasets, which introduces potential generalizability bias. In the global HIMALAYA cohort, patients presented with diverse etiologies, including hepatitis C virus (HCV) infection and non-viral causes. In contrast, approximately 80% of Chinese hepatocellular carcinoma (HCC) cases are driven by chronic hepatitis B virus (HBV) infection, which is frequently associated with more severe underlying post-hepatitic cirrhosis, impaired functional liver reserve, and distinct tumor immune microenvironments. These baseline clinical discrepancies may affect the direct transferability of trial efficacy and safety profiles to routine clinical practice in China. To bridge this gap, subsequent real-world pharmacoeconomic studies and pragmatic registry analyses are warranted to validate our model projections and further evaluate subgroup cost-effectiveness stratified by viral etiology (HBV vs. HCV/non-viral), baseline liver function (e.g., Child-Pugh and ALBI grades), and PD-L1 expression, thereby informing individualized clinical decision-making in Chinese tertiary oncology centers [37]. Second, health-related quality of life estimates associated with disease states were sourced from published literature, but these values did not incorporate utility decrements attributable to treatment-related adverse events (AEs). This omission may lead to an overestimation of quality-adjusted life years (QALYs), particularly for regimens with distinct AE profiles. In our model, post-progression therapy costs were accounted for based on trial proportions; however, the real-world management of advanced HCC is rapidly evolving toward novel sequential strategies and immune checkpoint inhibitor rechallenge [38]. Emerging evidence suggests that subsequent systemic therapy selection and ICI rechallenge upon progression can significantly alter post-progression survival trajectories and downstream medical expenditures. Future pharmacoeconomic models should incorporate dynamic, multi-line sequential treatment pathways to capture the comprehensive lifetime value of frontline immuno-oncology regimens. Third, although parametric survival curves (fitted using lognormal distributions) demonstrated close alignment with the index trial’s Kaplan-Meier data, the extrapolation of long-term survival outcomes remains inherently uncertain. To address this, we conducted extensive sensitivity analyses, including deterministic one-way sensitivity analyses and probabilistic sensitivity analyses with 10,000 Monte Carlo simulations. These analyses consistently demonstrated that STRIDE failed to achieve cost-effectiveness across plausible ranges of model parameters at conventional willingness-to-pay thresholds. Nevertheless, structural uncertainties in survival modeling highlight the need for validation using longer-term follow-up data. Fourth, in the Markov model formulation, the probability of dying directly from the progression-free state was assumed to reflect background mortality due to data reporting constraints. In advanced HCC, acute liver failure or variceal bleeding may cause mortality prior to confirmed disease progression. Although our dual-model design (PartSA vs. Markov) yielded nearly identical ICERs—indicating that this parameterization did not compromise our primary conclusions—future analyses with access to granular patient-level trial data should incorporate cause-specific pre-progression mortality rates.

## Conclusion

Based on the results of the base case analysis and sensitivity analysis, from the perspective of China’s healthcare system, the STRIDE program is not cost-effective when the willingness-to-pay threshold is set at three times the gross domestic product. Additionally, if the model is constructed appropriately, the results of the Markov model and the PS model are similar. This finding can provide guidance for Chinese doctors and the government.

## Supplementary Information

Below is the link to the electronic supplementary material.

Supplementary Material 1

## Data Availability

Data Availability Any data not provided in this publication can be provided from the corresponding author on reasonable request.

## Code availability

All data generated or analyzed during this study are included in this published article and its supplementary files.

## References

[1] Sung H, Ferlay J, Siegel RL, et al. Global cancer statistics 2020: GLOBOCAN estimates of incidence and mortality worldwide for 36 cancers in 185 countries. CA Cancer J Clin. 2021;71(3):209–49. 10.3322/caac.21660 33538338

[2] Abou-Alfa GK, Lau G, Kudo M, et al. Tremelimumab plus durvalumab in unresectable hepatocellular carcinoma. NEJM Evid. 2022;1(8):EVIDoa2100070. 10.1056/EVIDoa2100070 38319892

[3] Llovet JM, Castet F, Heikenwalder M, et al. Immunotherapies for hepatocellular carcinoma. Nat Rev Clin Oncol. 2022;19(3):151–72. 10.1038/s41571-021-00573-2 34764464

[4] Rongshou Z. Analysis of the prevalence of malignant tumors in China in 2022. Chin J Oncol. 2024;46(3):221–31.

[5] Han B, Zheng R, Zeng H, et al. Cancer incidence and mortality in China, 2022. J Natl Cancer Cent. 2024;4(1):47–53. 10.1016/j.jncc.2024.01.006 PMC11256708 39036382

[6] Wang Q-B, Luo W-L, Yang Z-S, et al. Reconsidering vascular invasion terminology in hepatocellular carcinoma staging: implications for accurate prognosis and treatment. J Natl Cancer Cent. 2026;6(2):120–1. 10.1016/j.jncc.2026.01.003 PMC13083625 42007216

[7] Rui M, Li H. Cost-effectiveness of osimertinib vs docetaxel-bevacizumab in third-line treatment in EGFR T790M resistance mutation advanced non-small cell lung cancer in China. Clin Ther. 2020;42(11):2159–e21706. 10.1016/j.clinthera.2020.08.018 33028495

[8] Desai A, Gyawali B. Financial toxicity of cancer treatment: Moving the discussion from acknowledgement of the problem to identifying solutions. Eclinicalmedicine. 2020;20:100269. 10.1016/j.eclinm.2020.100269 PMC7152810 32300733

[9] Yang C, Zhang H, Zhang L, et al. Evolving therapeutic landscape of advanced hepatocellular carcinoma. Nat Rev Gastroenterol Hepatol. 2023;20(4):203–22. 10.1038/s41575-022-00704-9 36369487

[10] Smare C, Lakhdari K, Doan J, et al. Evaluating partitioned survival and markov decision-analytic modeling approaches for use in cost-effectiveness analysis: Estimating and comparing survival outcomes. PharmacoEconomics. 2020;38(1):97–108. 10.1007/s40273-019-00845-x PMC7081655 31741315

[11] Aaronson NK, Ahmedzai S, Bergman B, et al. The european organization for research and treatment of cancer QLQ-C30: A quality-of-life instrument for use in international clinical trials in oncology. J Natl Cancer Inst. 1993;85(5):365–76. 10.1093/jnci/85.5.365 8433390

[12] Liao W, Huang J, Hutton D, et al. Cost-effectiveness analysis of cabozantinib as second‐line therapy in advanced hepatocellular carcinoma. Liver Int. 2019;39(12):2408–16. 10.1111/liv.14257 31544330

[13] Cranmer H, Shields GE, Bullement A. A comparison of partitioned survival analysis and state transition multi-state modelling approaches using a case study in oncology. J Med Econ. 2020;23(10):1176–85. 10.1080/13696998.2020.1796360 32673128

[14] Pichon-Riviere A, Drummond M, Palacios A, et al. Determining the efficiency path to universal health coverage: Cost-effectiveness thresholds for 174 countries based on growth in life expectancy and health expenditures. Lancet Glob Health. 2023;11(6):e833–42. 10.1016/S2214-109X(23)00162-6 37202020

[15] Cammà C, Cabibbo G, Petta S, et al. Cost-effectiveness of sorafenib treatment in field practice for patients with hepatocellular carcinoma. Hepatology. 2013;57(3):1046–54. 10.1002/hep.26221 23299720

[16] Husereau D, Drummond M, Augustovski F, et al. Consolidated health economic evaluation reporting standards 2022 (CHEERS 2022) statement: Updated reporting guidance for health economic evaluations. BMC Health Serv Res. 2022;22(1):114. 10.1186/s12913-021-07460-7 PMC8793223 35081957

[17] Williams C, Lewsey JD, Briggs AH, et al. Cost-effectiveness analysis in R using a multi-state modeling survival analysis framework: A tutorial. Med Decis Mak. 2017;37(4):340–52. 10.1177/0272989X16651869 PMC5424858 27281337

[18] Guyot P, Ades AE, Ouwens MJNM, et al. Enhanced secondary analysis of survival data: Reconstructing the data from published kaplan-meier survival curves. BMC Med Res Methodol. 2012;12:9. 10.1186/1471-2288-12-9 PMC3313891 22297116

[19] Plana D, Fell G, Alexander BM, et al. Cancer patient survival can be parametrized to improve trial precision and reveal time-dependent therapeutic effects. Nat Commun. 2022;13(1):873. 10.1038/s41467-022-28410-9 PMC8847344 35169116

[20] Jackson C. flexsurv: A platform for parametric survival modeling in R. J Stat Softw. 2016;70(8):1–33. 10.18637/jss.v070.i08 PMC5868723 29593450

[21] Liu L, Wang L, Ding Y, et al. Cost-effectiveness of atezolizumab plus bevacizumab versus sorafenib as first-line therapy in unresectable hepatocellular carcinoma in the US and Chinese setting: A modelling comparison study. BMJ Open. 2025;15(3):e094804. 10.1136/bmjopen-2024-094804 PMC11887288 40050065

[22] Zhou Ting M. Application of survival analysis in the calculation of transition probabilities in markov models for pharmacoeconomic evaluation. Chin J Evidence-Based Med. 2018;18(10):1129–34.

[23] Péter S, Eggersdorfer M, Van Asselt D, et al. Selected nutrients and their implications for health and disease across the lifespan: A roadmap. Nutrients. 2014;6(12):6076–94. 10.3390/nu6126076 PMC4277016 25533014

[24] Wen F, Zheng H, Zhang P, et al. Atezolizumab and bevacizumab combination compared with sorafenib as the first-line systemic treatment for patients with unresectable hepatocellular carcinoma: A cost‐effectiveness analysis in China and the united states. Liver Int. 2021;41(5):1097–104. 10.1111/liv.14795 33556230

[25] Wen F, Huang P, Wu Q, et al. Promising first-line immuno‐combination therapies for unresectable hepatocellular carcinoma: A cost‐effectiveness analysis. Cancer Med. 2024;13(16):e70094. 10.1002/cam4.70094 PMC11327610 39149756

[26] Zhang P-F, Xie D, Li Q. Cost–effectiveness analysis of nivolumab in the second-line treatment for advanced esophageal squamous cell carcinoma. Future Oncol. 2020;16(17):1189–98. 10.2217/fon-2019-0821 32407173

[27] Xu Z, Ye Z-M, Tang Y-K, et al. Cost-effectiveness analysis of sintilimab plus IBI305 versus sorafenib for unresectable hepatic cell carcinoma in China. Cancer Med. 2023;12(14):14871–80. 10.1002/cam4.5724 PMC10417160 37434398

[28] Rui M, Shi F, Shang Y, et al. Economic evaluation of cisplatin plus gemcitabine versus paclitaxel plus gemcitabine for the treatment of first-line advanced metastatic triple-negative breast cancer in China: Using markov model and partitioned survival model. Adv Ther. 2020;37(9):3761–74. 10.1007/s12325-020-01418-7 32647912

[29] Rui M, Wang Y, Fei Z, et al. Will the markov model and partitioned survival model lead to different results? A review of recent economic evidence of cancer treatments. Expert Rev Pharmacoeconomics Outcomes Res. 2021;21(3):373–80. 10.1080/14737167.2021.1893167 33691544

[30] Abou-Alfa GK, Lau G, Kudo M, et al. Plain language summary of the HIMALAYA study: Tremelimumab and durvalumab for unresectable hepatocellular carcinoma (liver cancer). Future Oncol. 2023;19(38):2505–16. 10.2217/fon-2023-0486 PMC13248070 37671641

[31] Liao W, Xu H, Hutton D, et al. Cost-effectiveness analysis of durvalumab plus tremelimumab as first-line therapy in patients with unresectable hepatocellular carcinoma. Ther Adv Med Oncol. 2024;16:17588359241274625. 10.1177/17588359241274625 PMC11412210 39301138

[32] Patel SP, Kurzrock R. PD-L1 expression as a predictive biomarker in cancer immunotherapy. Mol Cancer Ther. 2015;14(4):847–56. 10.1158/1535-7163.MCT-14-0983 25695955

[33] Zhang H, Dai Z, Wu W, et al. Regulatory mechanisms of immune checkpoints PD-L1 and CTLA-4 in cancer. J Exp Clin Cancer Res: CR. 2021;40(1):184. 10.1186/s13046-021-01987-7 PMC8178863 34088360

[34] Everest L, Shah M, Chan KKW. Comparison of long-term survival benefits in trials of immune checkpoint inhibitor vs non-immune checkpoint inhibitor anticancer agents using ASCO value framework and ESMO magnitude of clinical benefit scale. JAMA Netw Open. 2019;2(7):e196803. 10.1001/jamanetworkopen.2019.6803 PMC6624800 31290990

[35] Severens JL, Milne RJ. Discounting health outcomes in economic evaluation: The ongoing debate. Value Health: J Int Soc Pharmacoeconomics Outcomes Res. 2004;7(4):397–401. 10.1111/j.1524-4733.2004.74002.x 15449631

[36] Zhu Jiaxin. Evaluation of the value of precision medicine in tumor immunotherapy based on tumor mutation burden detection [Internet]. [GuangZhou]: Jinan University; 2022 [cited 2025 July 1]. Available from: 10.27167/d.cnki.gjinu.2021.000460

[37] Diaby V, Adunlin G, Montero AJ. Survival modeling for the estimation of transition probabilities in model-based economic evaluations in the absence of individual patient data: A tutorial. PharmacoEconomics. 2014;32(2):101–8. 10.1007/s40273-013-0123-9 24338265

[38] Li J, Liang Y-B, Wang Q-B, et al. Rechallenge with immune checkpoint inhibitors in patients with hepatocellular carcinoma: a narrative review. Liver Cancer. 2025;14(2):117–36. 10.1159/000543666 PMC12707946 41409409

